# Superplastic Behavior and Microstructural Features of the VT6 Titanium Alloy with an Ultrafine-Grained Structure during Upsetting

**DOI:** 10.3390/ma16041439

**Published:** 2023-02-08

**Authors:** Grigory S. Dyakonov, Andrey G. Stotskiy, Iuliia M. Modina, Irina P. Semenova

**Affiliations:** Laboratory of Multifunctional Materials, “Higher Engineering School of Aerospace Materials” Center, Ufa University of Science and Technology, 32 Zaki Validi St., 450076 Ufa, Russia

**Keywords:** titanium alloys, VT6, ultrafine-grained structure, severe plastic deformation, superplasticity, strain rate, sensitivity coefficient m, α_2_-phase

## Abstract

In this paper, the superplastic behavior of the two-phase titanium alloy VT6 with an ultrafine-grained (UFG) structure produced by equal-channel angular pressing is examined. The deformation of specimens with a UFG structure was performed by upsetting in a temperature range of 650–750 °C and strain rate range of 1 × 10^−4^–5 × 10^−1^ s^−1^. Under these conditions, an increased strain-rate sensitivity coefficient m was observed. The calculation of apparent activation energy showed values in a range of 160–200 kJ/mol while the superplastic flow of the VT6 alloy was occurring. When superplastic behavior (SPB) was impeded, the energy Q grew considerably, indicating a change in mechanism from grain-boundary sliding (GBS) to bulk diffusion. A change in temperature and strain rate influenced the development of superplastic flow and the balance of relaxation processes. Microstructural analysis shows that the UFG state is preserved at upsetting temperatures of 650 and 700 °C. A decrease in strain rate and/or an increase in upsetting temperature promoted a more active development of recrystallization and grain growth, as well as α_2_-phase formation. In a certain temperature and strain-rate range of the UFG VT6 alloy, α_2_-phase plates were found, the formation of which was controlled by diffusion. The effect of the α_2_-phase on the alloy’s mechanical behavior is discussed.

## 1. Introduction

The two-phase (α + β) titanium alloy VT6 is widely applied in aviation, e.g., in the manufacture of gas-turbine engine (GTE) disks and blades. These parts often experience high static and dynamic loads [1,2,3,4], which primarily requires high specific strength and hot strength. Among the methods that enable the mechanical properties of metals and alloys to be increased are various deformation and thermal treatments. One promising approach is the formation of an ultrafine-grained (UFG) state by severe plastic deformation (SPD) processing, which enables the constructural strength and operational characteristics of metals and alloys to be increased [5,6,7,8,9,10], which may be quite relevant for the parts of advanced GTEs.

The superplastic behavior of metals and alloys is normally studied by tensile tests at the corresponding temperatures and strain rates. This enables the stress–strain rate dependence to be evaluated, as well as an elongation to failure of several hundred percent. At the same time, the creation of a finished product normally implies several process operations, one of which is most often is shape forming by compression (die forging), which is conducted at rather high temperatures [11,12,13]. However, as noted by many researchers, UFG materials exhibit a higher technological plasticity due to the occurrence of the superplasticity effect at lower temperatures than in the case of standard deformation treatments [14,15,16]. As is widely known [11,17,18], products manufactured under the superplastic flow of a material exhibit homogeneity of the micro- and macrostructure in the whole product volume, which contributes to a high reliability of the product and a uniform distribution of stress in the material’s volume. However, the UFG structures formed in materials and, consequently, the produced properties are structurally sensitive characteristics, and the process of shape forming of parts at conventional temperatures may lead to their degradation. In this connection, an important task of the present work is to determine the temperature and strain-rate conditions for the ultrafine-grained titanium alloy VT6 under which the occurrence of the superplasticity effect and the preservation of structure in a UFG range as a primary indicator of the alloy’s mechanical characteristics are possible.

## 2. Materials and Methods

**Figure 1:**
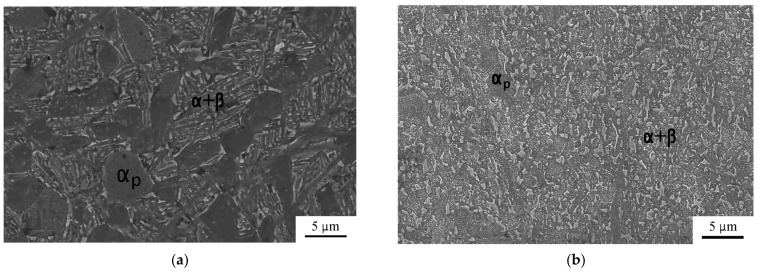
Microstructure of the VT6 alloy after HT (**a**) and after ECAP (**b**).

In this study, the VT6 titanium alloy was used in the form of rods with a diameter of 20 mm. VT6 is a Russian analogue of the Ti-6Al-4V alloy. The chemical composition of the alloy was found by optical emission spectrometry to be Ti-6.8%Al-4.3%V. The billets were subjected to a preliminary heat treatment (HT) via the following regime: water quenching at T = 960 °C for 1 h, aging at T = 675 °C for 4 h with air cooling. The microstructure of the alloy in the coarse-grained state represented a globular-lamellar structure (Figure 1a). The equal-channel angular pressing of the billets after HT was performed via the following regime: 4 passes via route C_b_ at T = 700 °C with a channel intersection angle of 120°, i.e., 180° rotation after the first pass, 90° rotation after the second pass, and 180° rotation after the third pass (0°–180°–90°–180°). After ECAP processing the VT6 alloy’s microstructure represented the partially preserved primary α-phase and partially globularized (α + β) particles with a size of about 0.2 μm (Figure 1b). According to the BSE analysis of the VT6 alloy [19], the β-phase is a bright phase. The average size of the β-phase particles was evaluated from the SEM photographs. The average grain size was determined following the standard procedure by the linear-intercept method.

The upsetting of the cylindrical specimens 6 × 7 mm in size with rates of 5 × 10^−1^, 5 × 10^−2^, 1 × 10^−2^, 5 × 10^−3^, 1 × 10^−3^, 5 × 10^−4^, 1 × 10^−4^ s^−1^ was conducted at temperatures of 650, 700 and 750 °C using an Instron 5982 universal testing machine (Instron Engineering Corporation, Buckinghamshire, UK). The microstructure of the specimens was studied using a Tescan Mira scanning electron microscope (TESCAN, Brno, Czech Republic) at an accelerating voltage of 20 keV. The fine electron microscopic studies of the specimens were performed using a JEM 2100 transmission electron microscope (JEOL, Tokyo, Japan) at an accelerating voltage of 200 kV. The strain-rate sensitivity coefficient was calculated according to the following formula:

(1) $$m=\partial \mathrm{lg}\left(\sigma \right)/\partial \mathrm{lg}\left(\xi \right)$$

The apparent activation energy was calculated according to the formula:

(2) $$Q=R/m\times \partial \mathrm{lg}\left(\sigma \right)/\partial \left(1/T\right).$$

## 3. Results

### 3.1. Mechanical Behavior during Upset

**Figure 2:**
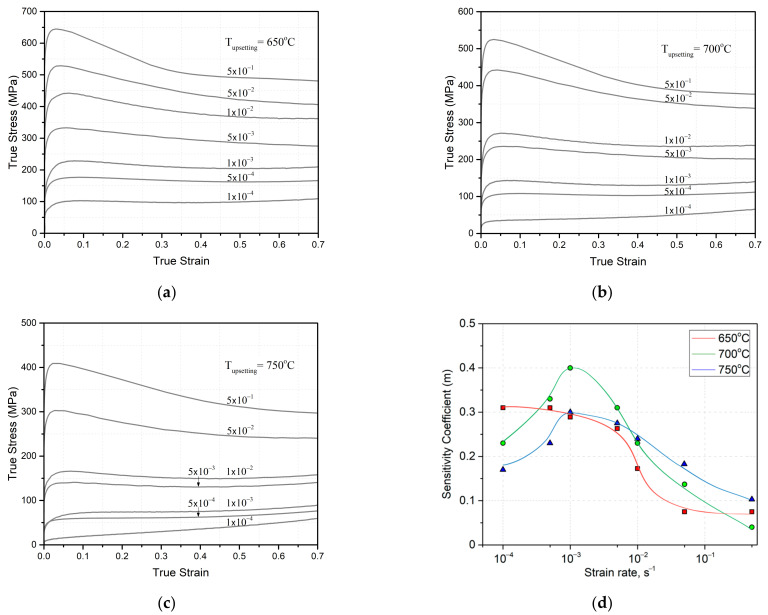
True stress–true strain curves after upset at temperatures of 650 °C (**a**), 700 °C (**b**), 750 °C (**c**), the strain-rate sensitivity coefficient m (**d**).

As a result of the upset of the UFG VT6 alloy at temperatures (650, 700, 750 °C) in a strain rate range of 5 × 10^−1^× 10^−4^ s^−1^, true stress–true strain curves were constructed (Figure 2a–c).

In the diagrams obtained during the upset of the alloy, high strain rates (5 × 10^−1^–1 × 10^−2^ s^−1^) were characterized by an intensive increase in flow stress in the beginning, and furthermore, the stress value decreased with increasing strain.

Such strain hardening at the initial stage of deformation (at e < 0.1) is ensured by a strong increase in dislocation density [20]. During the further upset of the UFG VT6 alloy (at e > 0.1), a decline in flow stress is observed. This is conditioned by the relaxation of internal stresses in the process of dynamic recovery and the activation of grain-boundary sliding, which are characteristic of superplasticity.

At relatively low strain rates (1 × 10^−3^ s^−1^–1 × 10^−4^ s^−1^), the view of the curves changes. The curves practically immediately reach a horizontal plateau—stress practically does not vary. It is clearly visible that an increase in the upsetting temperature provides a decrease in stress during upset.

Analysis of the strain-rate sensitivity coefficient m shows that the maximum value of the coefficient m ~ 0.4 can be observed at T = 700 °C and a strain rate of ~1 × 10^−3^ s^−1^ (Figure 2d). An increase or decrease in strain rate at T = 700 °C leads to a decrease in the value of the coefficient m. A similar character of variation of the coefficient m was obtained for deformation at T = 750 °C. The difference was that at a deformation temperature of 750 °C, the maximum coefficient m was equal to 0.3.

A totally different character of variation of the coefficient m was observed at a deformation temperature of 650 °C. In the region of low strain rates (~10^−4^ s^−1^), the coefficient m reached its maximum, ~0.32. Increasing strain rate was accompanied by a decrease in the coefficient m, and an especially drastic decrease in the coefficient m took place at a rate of 10^−2^ s^−1^ (Figure 2d).

### 3.2. Evolution of the Microstructure during Upset

**Figure 3:**
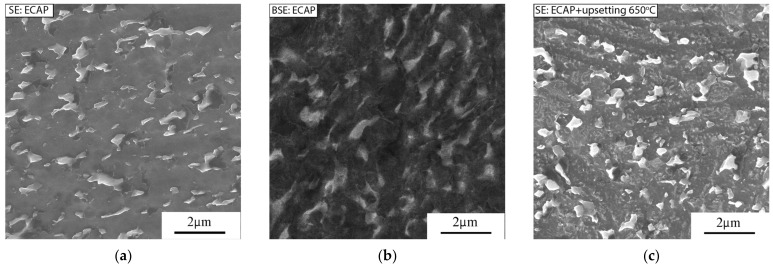

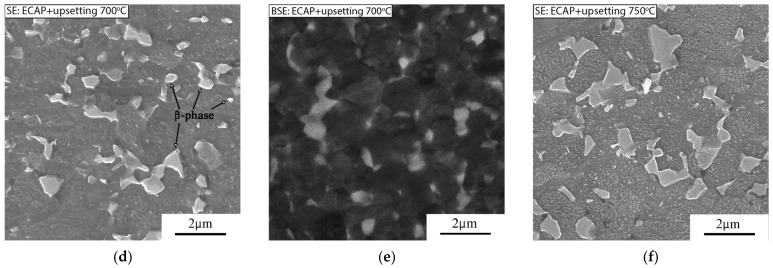
Microstructure of the VT6 alloy after ECAP (**a**,**b**) and after additional upset with a strain rate of 1 *×* 10^−3^ s^−1^ at the following temperatures: (**c**) 650 °C, (**d**,**e**) 700 °C, (**f**) 750 °C (SE and BSE detectors).

The microstructural evolution of the UFG VT6 alloy during upset (e = 0.7) at a strain rate of 1 *×* 10^−3^ s^−1^ is shown in Figure 3. After ECAP processing, a slight elongation of grains in the direction of shear is observed (Figure 3a,b). The size of the β-phase particles in the ECAP-processed material is 0.22 ± 0.02 μm. After upset at 650 °C, a slight spheroidization of the β-phase particles to sizes of 0.25 ± 0.03 μm was observed (Figure 3c). The BSE analysis after ECAP processing and an additional upsetting at 700 °C confirmed the morphology of the β-phase and revealed a gradual globularization and coarsening of the β-phase particles (Figure 3b,e). With the temperature increasing to 700 and 750 °C, it can be seen that the β-phase particles globularize more fully and grow to 0.3 and 0.4 μm, respectively (Figure 3d,f).

**Figure 4:**
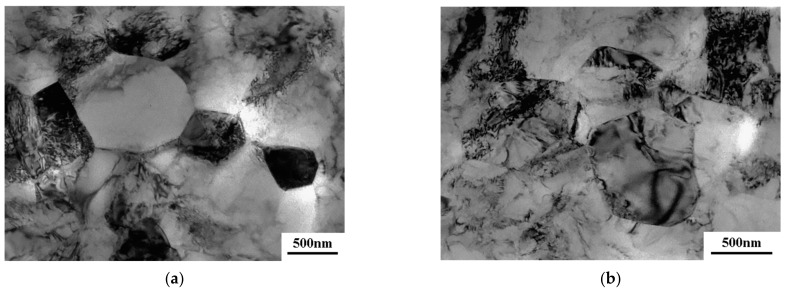
Microstructure of the VT6 alloy after ECAP (**a**) and after upset with a strain rate of 5 *×* 10^−4^ s^−1^ at a temperature of 650 °C (**b**).

A more detailed study of the microstructural evolution of the UFG alloy was conducted on a transmission electron microscope. After ECAP processing, the microstructure represents a mixture of grains and subgrains, and a relatively high dislocation density is observed in the interiors of separate grains. At the same time, well visible are the clear boundaries and triple junctions of grains. This structure is typical for the VT6 titanium alloy after SPD processing (Figure 4a). In the ECAP-processed sample, the average grain size is 530 ± 30 nm. The upset of the UFG VT6 alloy at T = 650 °C resulted in a slight increase in the average grain size (taking into account the α- and β-phases) to 620 ± 40 nm. It can be seen that grain boundaries bend, and in the near-boundary region the dislocation density is increased.

Comparing the results of TEM and mechanical tests (Figure 2d), it can be asserted that at an upset temperature of 650 °C and a strain rate of 5 × 10^−4^ s^−1^ the microstructural evolution is controlled by the traditional mechanism due to dislocation slip, dynamic recovery and continuous dynamic recrystallization. Analyzing the character of the microstructure and the value of the coefficient m (close to 0.3), these deformation conditions (650 °C and 5 × 10^−4^ s^−1^) can be identified as the boundary conditions for the occurrence of superplasticity.

**Figure 5:**
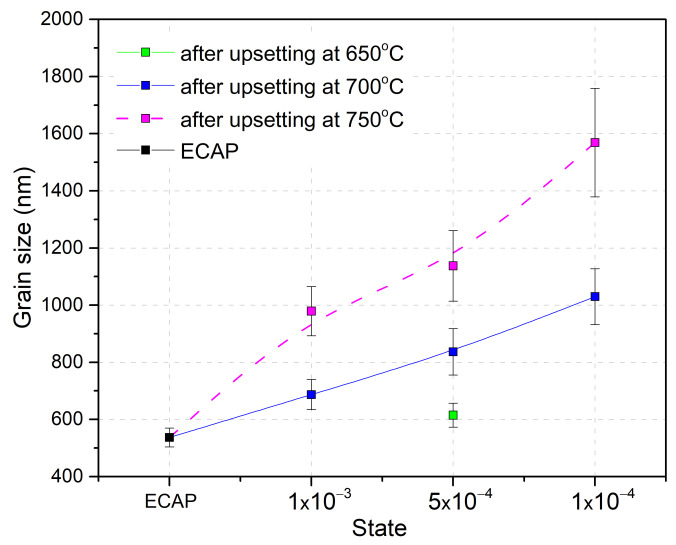
Variation in the mean grain size of the UFG VT6 alloy after upset, depending on temperature and strain rate.

The variation in the mean grain size of the deformed samples with the deformation temperature increasing from 650 to 750 °C is shown in Figure 5**.** An increase in grain size is related to the development of recrystallization, the influence of which grows with decreasing strain rate.

**Figure 6:**
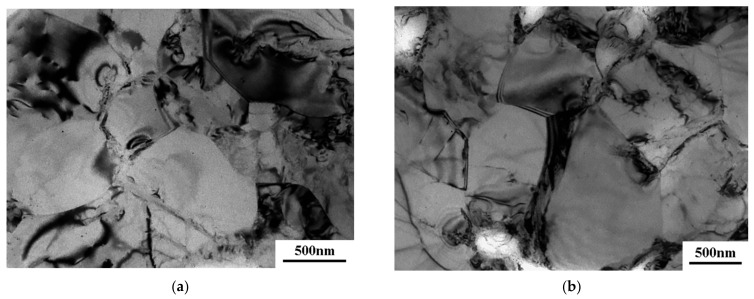

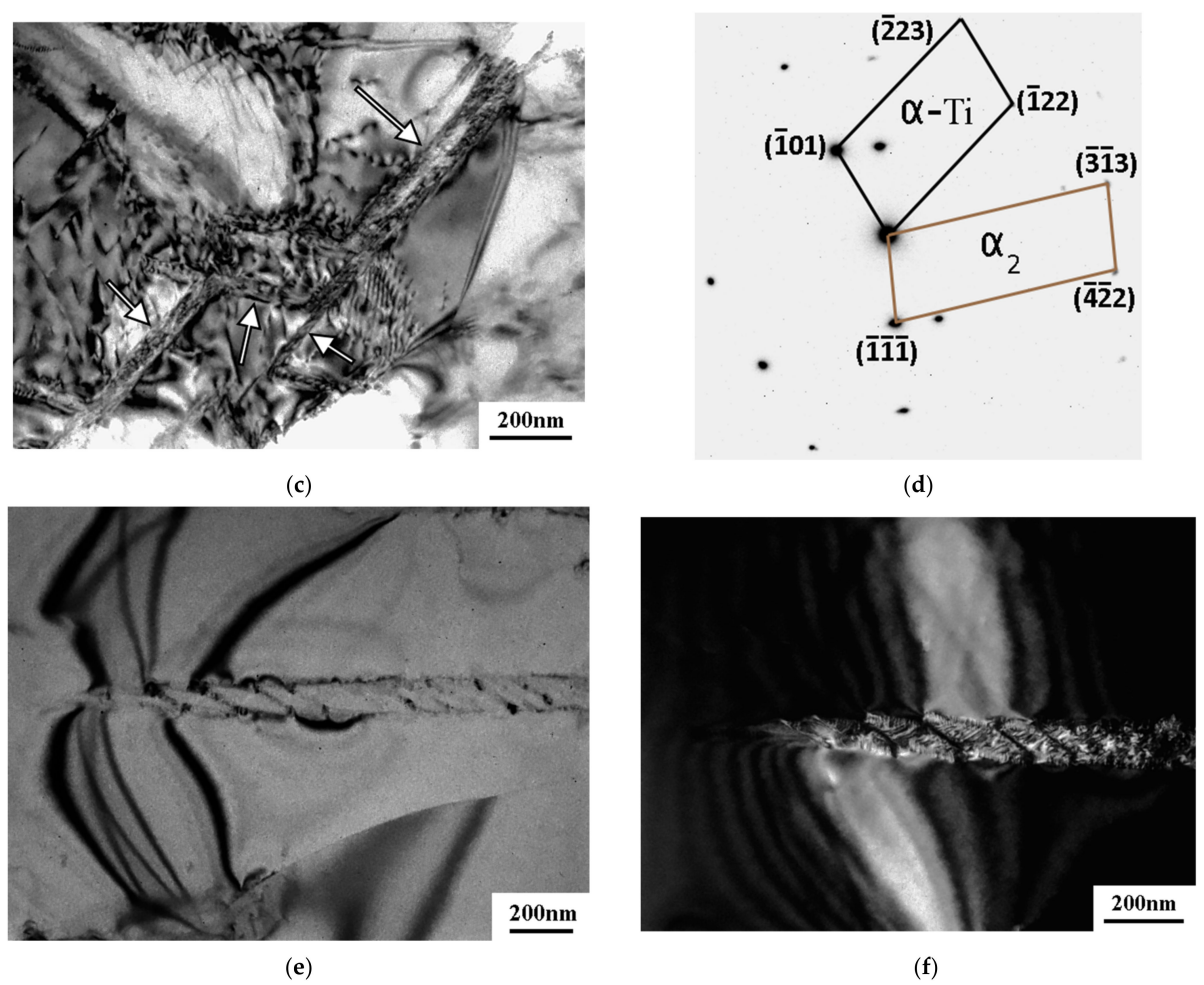
Microstructure of the VT6 alloy after upset at a temperature of 700 °C with strain rates of 1 *×* 10^−3^ s^−1^ (**a**), 5 *×* 10^−4^ s^−1^ (**b**,**c**), diffraction from a plate and a grain (**d**), and with a strain rate of 1 *×* 10^−4^ s^−1^ (**e**,**f**).

Microstructural study after upset at 700 °C and strain rates of 1 *×* 10^−3^ and 5 *×* 10^−4^ s^−1^, when the coefficient m reached its highest values, revealed that the grains were practically free from dislocations (Figure 6a,b). That being said, an increase in the deformation temperature to 700 °C led to an increase in the mean grain size to 680 ± 50 nm, and at 5 *×* 10^−4^ s^−1^ ~ 840 ± 80 nm.

In separate grains, plates were discovered, no more than 50 nm thick (Figure 6c, as marked by arrows), that intersected with the grain and sometimes intersected with each other. The formation of plates in grain interiors leads to the emergence of additional boundaries that may act as an obstacle for dislocation motion (Figure 6c). The precise diffraction analysis of the matrix and plates shows that these are α_2_-phase plates (Figure 6d).

Upset at T = 700 °C at a lower strain rate of ~1 *×* 10^−4^ s^−1^ was accompanied by an increase in the mean grain size to 1 μm due to the development of recrystallization. As in the previous case, α_2_ plates were discovered in separate grains. However, the morphology of these plates was different. Firstly, the plate thickness increased to 100 nm, and secondly, these plates contained in their interiors smaller lens-like lamellae (Figure 6e,f). At higher strain rates, there were no α_2_ plates revealed in the microstructure.

**Figure 7:**
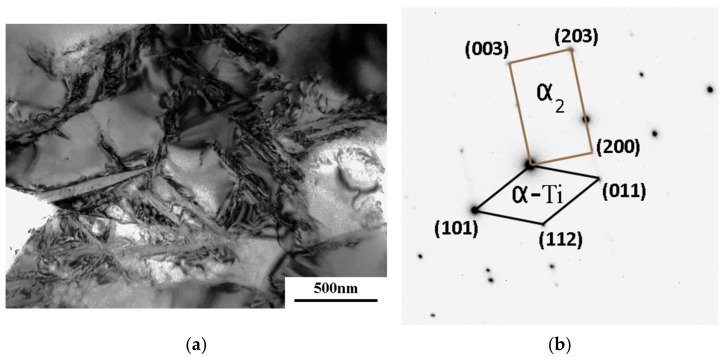
Microstructure of the VT6 alloy after upset at a temperature of 750 °C with a strain rate of 1 *×* 10^−3^ s^−1^ (**a**), diffraction from a plate and a grain (**b**).

An increase in the upset temperature to 750 °C resulted in an increase in the mean grain size to 1–1.5 μm at strain rates of 1 *×* 10^−3^–1 *×* 10^−4^ s^−1^. At the same time, it was found that at such high temperature α_2_-phase plates formed even at a strain rate of 1 *×* 10^−3^ s^−1^ (Figure 7a,b). These results show that the formation of the α_2_-plates is a diffusion-dependent process that is activated with increasing deformation temperature.

## 4. Discussion

The superplasticity of metals and alloys is characterized by an increased strain-rate sensitivity coefficient m > 0.3 [17].

The ultrafine-grained structure in the VT6 alloy provided superplastic behavior at temperatures of 650–750 °C. For the VT6 alloy with a coarse-grained structure, the cases of grain-boundary sliding and large elongations under deformation are normally observed at 850 °C and above [1,17,21].

It is well known that the strain-rate sensitivity coefficient m depends on grain size, the volume fraction of a phase, deformation temperature, etc. [17,22,23]. Variation of the coefficient m and the results of microstructural studies indicate that at a relatively low deformation temperature of 650 °C the UFG VT6 alloy exhibits superplastic behavior only in the region of low strain rates ~10^−4^ s^−1^ (Figure 2d). An increase in the strain rate on the microstructural scale leads to a rapid growth of dislocation density, while the decelerated process of dynamic recovery does not provide the softening effect.

An increase in deformation temperature to 700 °C results in a different behavior of the coefficient m (Figure 2d). In the region of low strain rates ~10^−4^ s^−1^, one may call dynamic recovery and grain growth the controlling mechanisms; this leads to the relatively low values of m ~ 0.2. Additionally, α_2_-phase plates that act as an additional obstacle for dislocation motion and annihilation are found in the structure. The formation of the α_2_-phase in the VT6 alloy may take place during aging or after certain heat treatments, and very often it precipitates in the form of fine dispersed particles in a grain body [24,25,26]. In addition, the thermodynamic calculation performed by the authors in [27] confirms the possibility of the formation of the α_2_-phase in the VT6 alloy. The formation of the α_2_-phase in the form of very fine plates was experimentally confirmed during the isothermal deformation of the VT6 alloy at a strain rate of 0.01 s^−1^ and in the deformation temperature range of 650−1000 °C [27]. Studies have shown that the α_2_ phase may form via both the spinodal mechanism and the nucleation mechanism [24,25,26,28].

An increase in strain rate to ~10^−3^ s^−1^ at 700 °C leads to a balance between the processes of dislocation formation and annihilation. The TEM studies and a large number of grains free from dislocations confirm this and indicate the activation of grain-boundary sliding characteristic of superplastic behavior. A further increase in strain rate of ~10^−2^ s^−1^ upsets the balance, the microstructure is characterized by an increased dislocation density, and the coefficient m approaches the value of 0.2.

Deformation at 750 °C demonstrates the relatively low values of the coefficient m. In the region of low strain rates ~10^−4^ s^−1^ the mean grain size increases 3-fold and reaches sizes of ~1.5 μm. Inside separate grains, α_2_-phase plates are observed. As strain rate increases to ~10^−3^ s^−1^, the coefficient m in the diagram barely touches the value of 0.3. According to our expectations, this value could even be slightly higher than 0.3, but this was apparently prevented by grain growth to 1 μm and the presence of α_2_-phase plates in separate grains. As a result, this impeded the superplastic behavior of the UFG alloy.

Thus, one can outline several characteristic areas that characterize processes occurring during the upsetting of the UFG VT6 alloy.

**Figure 8:**
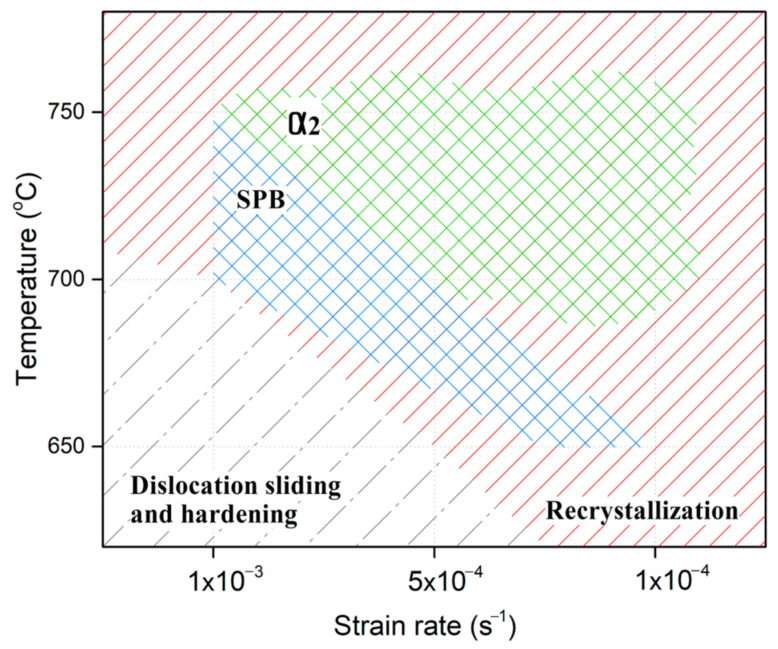
Schematic presentation of the processes occurring during upset, depending on temperature and strain rate.

When high strain rates and low temperatures are combined, deformation is realized via the traditional mechanism due to dislocation slip, which is accompanied by strengthening (the gray area in Figure 8).

An increase in temperature and/or a decrease in strain rate activates the development of continuous dynamic recrystallization (the red area in Figure 8), and under certain temperature and strain-rate conditions, the alloy exhibits superplastic behavior (the blue area in Figure 8), while under other conditions, the largest contribution to the microstructural changes and mechanical behavior of the UFG VT6 alloy is made by dynamic recrystallization (the red area in Figure 8) and the formation of the α_2_-phase (the green area in Figure 8). The activation of continuous dynamic recrystallization is confirmed by the alloy’s microstructural changes and mechanical behavior.

**Figure 9:**
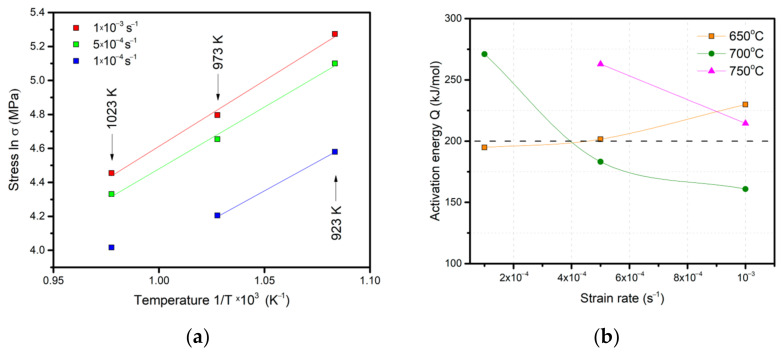
Plot of lnσ vs. 1/T (**a**) and the calculated values of apparent activation energy at the upsetting temperatures (**b**).

A plot of lnσ vs. 1/T was made, showing a linear dependence for the strain rates of 1 *×* 10^−3^ and 5 *×* 10^−4^ s^−1^ over the whole temperature range (Figure 9a). For 1 *×* 10^−4^ s^−1^, at a temperature of 750 °C, a distinct deviation from linearity is observed, which may be related to a change in the ratio between the α and β phases [22]. The calculation of apparent activation energy showed that at 650 °C the minimum value of Q = 195 kJ/mol corresponded to the rates of 2 *×* 10^−4^ s^−1^–4 *×* 10^−4^ s^−1^ (Figure 9b). The value of apparent activation energy during superplastic behavior is normally below 200 kJ/mol, whereas the value of apparent activation energy close to ~300 kJ/mol corresponds to the activation energy of bulk diffusion [15,22,29,30,31]. Based on the data from the calculation of the apparent activation energy and the coefficient m, the superplastic behavior of the UFG VT6 alloy at a temperature of 650 °C is limited to the strain rates of 1 *×* 10^−4^ s^−1^–5 *×* 10^−4^ s^−1^.

At 700°C, the values of apparent activation energy are located below 200 kJ/mol in a strain-rate range of 5 *×* 10^−4^ s^−1^–1 *×* 10^−3^ s^−1^, and this is also in good agreement with the values of the coefficient m. A decrease in strain rate 1 *×* 10^−4^ s^−1^ is accompanied by an increase in Q to 270 kJ/mol, which indicates a change in the controlling mechanisms of deformation, the domination of diffusion processes and the acceleration of structure recrystallization.

The evaluation of Q at 750 °C was performed only for the rates of 1 *×* 10^−3^ and 5 *×* 10^−4^ s^−1^. The calculation results showed 215 and 260 kJ/mol, respectively, which also indicates a transition from superplastic deformation to deformation controlled by continuous dynamic recrystallization, grain growth and the formation of α_2_-phase plates.

## 5. Conclusions

1. During the upset of the UFG VT6 alloy the temperature and strain-rate ranges of the superplasticity effect were found. At relatively low temperatures of T = 650 °C the UFG VT6 alloy exhibits superplastic behavior only at low strain rates of 1 *×* 10^−4^ s^−1^–5 *×* 10−4 s^−1^. An increase in the deformation temperature to 700 °C leads to the occurrence of superplasticity in a strain-rate range of 5 *×* 10^−4^ s^−1^–1 *×* 10^−3^ s^−1^. Deformation at T = 750 °C was to a greater extent controlled by the processes of dynamic recovery and continuous dynamic recrystallization.
2. As a result of the upset of the UFG alloy, the formation of the α_2_ phase in the form of plates was revealed. The temperature conditions for the formation of the α_2_-phase plates indicate that it is a diffusion-dependent process. Microstructural studies, mechanical test results and analysis of the coefficient m indicate that the α_2_-phase plates may act as an obstacle for dislocation motion and thereby ensure the alloy’s strengthening.
3. Calculation of apparent activation energy and analysis of the strain-rate sensitivity coefficient show that a range of 160–200 kJ/mol is characteristic of the superplastic behavior of the UFG VT6 alloy. An increase in the value of apparent activation energy above 200 kJ/mol was accompanied by a decrease in the coefficient m < 0.3 and indicated a change in the mechanism from GBS to bulk diffusion.

## Data Availability

Not applicable.
